# Perceived meaning of life and satisfaction with life: A research synthesis using an online finding archive

**DOI:** 10.3389/fpsyg.2022.957235

**Published:** 2023-02-10

**Authors:** Yomna Sameer, Yasmine Eid, Ruut Veenhoven

**Affiliations:** ^1^College of Business, Abu Dhabi University, Abu Dhabi, United Arab Emirates; ^2^University of London Recognized Teaching Center, Arab Academy for Science, Technology and Maritime Transport (AASTMT), Cairo, Egypt; ^3^Erasmus Happiness Economics Research Organization (EHERO), Erasmus University Rotterdam, Rotterdam, Netherlands; ^4^Opentia Research Program, North-West University, Vanderbijlpark, South Africa

**Keywords:** happiness, sense of mission, purpose in life, review, life-satisfaction, search for meaning, findings archive

## Abstract

**Introduction:**

“Meaning” and “happiness” are leading topics in positive psychology, but their relationship is not well understood. The first step to better understanding is to inspect the pattern of correlations found in the research literature. Specifically, we seek answers to the following questions of fact: (1) Is there a correlation between perceived meaning of life and satisfaction with life? (2) If so, is that correlation positive or negative? (3) How strong is this correlation? (4) How variable is this correlation across persons and situations? (5) Do the correlations differ across components of happiness? (6) What aspects of meaning are most/least associated with happiness? (7) What sources of meaning are most/least associated with happiness? (8) Does seeing meaning relate differently to happiness than searching for meaning?

**Method:**

We took stock of the available research findings, using the World Database of Happiness, which holds standardized descriptions of 171 observed relations between perceived meaning of life and satisfaction with life.

**Results:**

We found strong correlations between happiness and the degree of perceived meaning in life but little correlation with the pursuit of meaning. While the correlation with the degree of meaning is positive at the micro-level of individuals, it appears to be negative at the macro-level of nations.

**Discussion:**

Having established the above mentioned matters of fact, we considered the following questions on causality: (1) Is there an innate need for meaning? (2) How does the perceived meaning of life otherwise affect satisfaction with life? (3) How does satisfaction with life affect the perceived the meaning of life? (4) Why is the correlation positive at the micro-level of individuals, but negative at the macro-level of nations?

**Conclusions:**

We conclude that there is no innate human need for meaning. Still, the perceived meaning of life can affect life satisfaction in various other ways, while life satisfaction will also affect the sense of meaning. Both positive and negative effects can be involved, the balance of which tends to be positive for seeing meaning but close to neutral for pursuing meaning.

## Introduction

“Meaning” and “happiness” are leading topics in positive psychology, but their relationship is not well understood. Most attention has been devoted to the differences: meaning being presented as an aspect of *eudaimonic* happiness and happiness in the sense of life satisfaction as *hedonic* happiness. This conceptual difference goes often with a moral preference for the former over the latter, which fits the current practice in positive psychology, positive psychological interventions (PPIs), focusing more on strengthening eudaimonic strengths than on boosting life satisfaction. Although recognized as separate topics, the relationship between meaning and happiness is little understood.

### Views

Perceived meaning of life and satisfaction with life are both appraisals of the quality of life. Their relation can be considered from the following perspectives.

*Need-theory* of happiness holds that we feel better when innate needs are being fulfilled and that this affective experience gives rise to greater satisfaction with life (Veenhoven, 2009). One such innate need is presumed to be a need for meaning Steger and Frazier (2005); Routledge and FioRito (2021). If so, the perceived meaning of life will go together with satisfaction with life, and this correlation will be universal. A positive correlation can also be expected if meaning adds to happiness through the gratification of other needs, for instance, if a sense of mission pushes to active involvement in life and as such caters to needs for social respect and the use of one's potentials. In this instrumental perspective, one can also think of a negative effects of a sense of meaning on life satisfaction. A strong sense of mission can interfere with the gratification of other needs, such as in the case of Catholic priests who choose to forego sexual contact.

Next, there are several *cognitive views* on happiness, one of which holds that the satisfaction with one's life as a whole results from the summing of satisfactions with parts of life in a “bottom-up” process. Part satisfactions concern *domains* of life, such as family and work, as well as satisfaction with *aspects* of life, such as its richness or uniqueness (Andrews and Withey, 1976). In that context, the perceived meaning of life can be seen as an aspect evaluation of life. Seeing life as meaningful will then add to life satisfaction, while seeing life as meaningless will detract from it. In the cognitive view, satisfaction with that aspect of life will depend on the degree to which life fits a *want* for meaning, which can draw on an innate need for meaning but can also be a cultural phenomenon.

The effects of the perceived meaning of one's life on satisfaction with life are likely to vary across persons and situations. Contingencies will exist for the positive and negative effects and for the effect through gratification of innate needs as well as for meeting learned wants. One of the personal moderators will be the aptness to believe in a cause and the ability to cope with philosophical doubt about the meaning of life. Situational moderators will be in culture; a sense of meaning is more likely to add to the happiness in cultures that value living a meaningful life and provide practicable models to do so.

Next to the effects of perceived meaning on satisfaction with life, there can be effects of life satisfaction on perceived meaning in life. These “top-down” effects are also likely to be contingent on personal and situational characteristics.

### Questions on correlation

In this study, we started by answering some questions on basic facts.

- Is there a correlation between the perceived meaning of life and satisfaction with life?
- If so, is that correlation positive or negative?
- How strong is this correlation?
- How variable is this correlation across persons and situations?
- Do the correlations differ across components of happiness?
- What aspects of meaning are most/least associated with happiness?
- What sources of meaning are most/least associated with happiness?
- Does seeing meaning relate differently to happiness than searching for meaning?

We sought answers to these questions by taking stock of the available research findings. Selection of such findings required that we be clear about what we mean by “perceived meaning of life” and with “satisfaction with life” and that we establish how these phenomena can be measured. We did that in section “Concepts”. Next, we reviewed the available research findings on the relationship between the perceived meaning of life and happiness, drawing on a finding archive, the World Database of Happiness. We described that source in section “Method of this research synthesis” and reported the findings in section “Results”.

### Questions about causation

Having reviewed the observed correlations between meaning and happiness, we considered the following questions about the causality behind the relation between the perceived meaning of life and satisfaction with life in section “Discussion”:

- Is there an innate need for meaning?
- How does the perceived meaning of life otherwise affect satisfaction with life?
- How does satisfaction with life affect the perceived meaning of life?
- How strong are these effects relatively?
- Why is the correlation positive at the micro-level of individuals, but negative at the macro-level of nations?

### Difference from common reviewing

Our approach differs from common practice in research review, which starts with theoretical questions and next presents empirical findings that support or oppose hypotheses. The disadvantages of that procedure are: selective presentation of the available research findings, often involving “cherry picking”, and underreporting of the findings that do not fit a hypothesis.

In this study, we started taking stock of facts in section “Results” and next considered what these data tell about some questions about causation in the relation between the meaning of life and happiness in section “Discussion”. As the reader will see, this allows for a complete overview of the available research findings, which can easily be updated. This inductive approach reveals findings that are likely to escape theoretical imagination, such as in this case, that the correlation is positive at the micro-level of individuals but negative at the macro-level of nations.

## Concepts

In the widest sense, the word “happiness” is seen as denoting “living a good life”, while the term “meaning of life” is taken to refer to what a life contributes to something good beyond that life. As such, there is a conceptual overlap between these notions; meaning is part of happiness. A correlation between living a good life and living a meaningful life is therefore implied but can hardly be demonstrated empirically since we cannot measure how “good” a life is and neither how much “good” of a life contributes to other sakes than that life itself.

In this study, we focus on happiness and meaning in the more limited sense of subjective appraisals of one's life, respectively, with *perceived meaning of one's life* and *satisfaction with life*. These are measurable phenomena. We will further avoid the use of the words “meaning” and “happiness” here since these suggest a wider objective worth. We will deal with subjective perceptions of one's life and what we want to determine is how these subjective appraisals relate.

### Satisfaction with life

We follow the definition of life satisfaction as the *overall appreciation of one's life as a whole*; in other words, how much one likes the life one lives. This concept is at the basis of the World Database of Happiness, our data source. A detailed delineation is found here.

#### Components of life satisfaction

In assessing how much we like the life we live, we draw on two sources of information: how well we feel most of the time and the degree to which we perceive that life brings us what we want from it. These sub-appraisals are referred to as “components” of life satisfaction, which are an affective component called *hedonic level of affect* and a cognitive component called *contentment*. The differences between overall life satisfaction and these components are explained in more detail here. In this study, we explore whether the relationship with the perceived meaning of life differs across overall life satisfaction and these components.

#### Measures of life satisfaction

Since life satisfaction is something we have in mind, it can be measured using questioning. Some common questions are as follows:

- *Questions on overall life satisfaction*
  ° Taking all together, how happy would you say you are these days?
  ° On the whole, how satisfied are you with the life you lead?

- *Questions on hedonic level of affect*.
  ° Would you say that you are usually cheerful or dejected?
  ° During the past few weeks, did you ever feel....? (yes/no)^1^
    + Particularly excited or interested in something?
    – So restless that you couldn't sit long in a chair?
    + Proud because someone complimented you on
    something you had done?
    – Very lonely or remote from other people?
    + Pleased about having accomplished something?
    – Bored?
    + On top of the world?
    – Depressed or very unhappy?
    + That things were going your way?
    – Upset because someone criticized you?
    (Affect balance computed subtracting negative from
    positive “yes” responses)
  ° How is your mood today? (repeated several days)

- *Questions on contentment*
  ° How important was each of the following goals in life in the plans you made for yourself in early adulthood?
  ° How successful have you been in the pursuit of these goals?

### Perceived meaning of life

While life satisfaction is a rather clear concept (how much you like the life you live), the perceived meaning of life is a more ambiguous notion. The word “meaning” has different connotations, which cannot be captured in one distinct concept. This leaves us with a set of aspects of perceived meaningfulness, which can be considered separately or in sum.

#### Aspects of perceived meaning of life

When interpreted as an answer to the existential question of *why we live*, perceived meaning refers to the role of human life in evolution and one's place in that context. This involves philosophical reflection in which not everybody will engage. When interpreted as *what one lives for*, it refers to a sense of mission, such as a better life for one's children, which involves some good beyond one's own life. This is referred to as the perceived *usefulnes*s of one's life. However, the meaning of life is also seen in a *sense of direction*, which does not necessarily imply contribution to a greater good, such as getting rich. Other aspects or perceived meaning of life are the *significance* of one's life to the good or the bad or the *uniqueness* of one's life. These differences are reflected in the questions used in research on the perceived meaning of one's life.

Another topic that is often addressed in studies on perceived meaning is a *sense of coherence*. We see this as a matter of personality integration and will not consider it in this study.

#### Measures of perceived meaning of life

Like life satisfaction, perceived meaning in life can be measured using questioning, typically asking respondents to endorse or not statements such as the following:

- I feel my life is meaningful.
- My life has no clear purpose.

Note that these terms are not synonymous, meaning does do not always require a purpose (direction), and a purpose can be meaningless (useless). Next to such items on either meaning or purpose in questionnaires, several measures combine these aspects, either in one question or in multiple questions.

- My personal existence is utterly meaningless, without purpose.

Some questionnaires also contain items on other aspects of the meaningfulness set, such as these items in the Purpose in Life test (Crumbaugh and Maholick, 1969).

- In achieving life goals I've made no progress whatever (vs progressed to complete fulfillment).
- My life is: empty, filled only with despair (vs running over with exciting things).
- If I should die today, I'd feel that my life has been completely worthless (vs very worthwhile).
- In thinking of my life, I often wonder why I exist (vs always see reasons for being here).

The more aspects of meaning addressed in a questionnaire, the less clear it becomes what it measures. An additional problem for this study was that such questionnaires also contain items close to happiness, such as the item on suicidal ideation in the Purpose in Life test.

Next to the above questions on the *degree* of perceived meaning of life, there are also questions on the *satisfaction* with one's sense of meaning. These things do not necessarily go together; one can see little meaning in one's life but nevertheless be satisfied with that. Other aspects of perceived meaningfulness are the *search for meaning* and perceived *sources of meaning*.

All this presumes that people have an idea about the meaning of their life, which is not the case, at least not for young children. So, further variable aspects of the perceived meaning of life are the degree to which one *gives it a thought* and, if so, how much one is *concerned* with the issue.

## Method of this research synthesis

**Figure 1:**
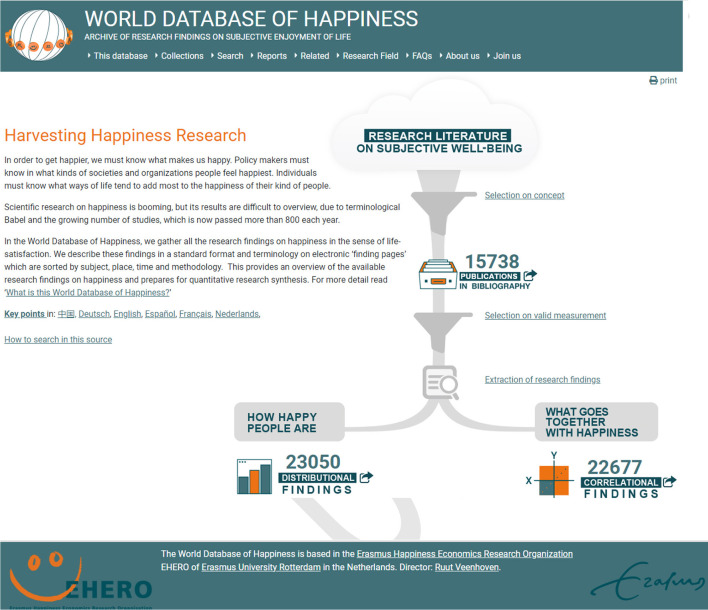
Homepage of the world database of happiness. https://worlddatabaseofhappiness.eur.nl. Source: World Database of Happiness. Reproduced with permission.

The first step in this review was to gather the available research findings on the relationship between the perceived meaning of one's life and satisfaction with life. The second step was to present these findings in an uncomplicated form. For both steps, we used the World Database of Happiness, which is an online “finding archive” on happiness in the sense of life satisfaction. The structure of this source is depicted visually in Figure 1, and a more detailed description is found here.

### Selection of studies

We could draw on an existing collection of reports of research on our topic, available in the collection of correlational findings of the World Database of Happiness, subject section Meaning of life. This collection is gathered using the following steps:

- Scientific publications on happiness are gathered continuously in the context of the World Database of Happiness. The selection criterion is that happiness in the sense of life satisfaction is addressed.
- Selected publications are included in the Bibliography of Happiness and their main topics are noted using a subject classification. One of the subject categories in the Bibliography is Perceived meaning of Life.
- Publications are selected from this collection that reports an empirical investigation using an acceptable measure of happiness. This procedure is described in more detail here.

We updated the collection, which left us by 30 April 2022 with 28 *publications*, which together reported 75 *studies* (one publication reported 47 studies). These studies are listed in Table 1. Together, they yield 171 separate *findings* on which we focus in this study.

**Table 1:** 75 studies in which a correlation between perceived meaning and life satisfaction was assessed.

**People**	**Measure of perceived meaning**	**Measure of happiness**	**Study excerpt in finding archive**	**Publication in reference list**
Leas				
**General public**				
18+ aged, USA. 201?	Three questions on perceived meaning	Time felt happy	US 2008	Baumeister et al., 2013
15+ aged, 94 nations, 2006–2007	Do you feel your life has an important purpose or meaning?	Best-worst possible life	ZZ 2006	Joshanloo, 2018
16+ aged general public, The Netherlands 2020	Goal directedness Existential significance	Happiness	NL 2020	DeHart et al., 2022
18+ aged, 85 developed and developing countries, 199-−2007	How often, if at all, do you think about the meaning and purpose of life?	Happiness	ZZ 1994	Duff and Ivlevs, 2011
18+ aged, general public, USA, 1973/7	How do you feel about how much you are really contributing to other people's lives?	Life-satisfaction (question asked twice)	US 1973/1	Andrews and Withey, 1976
15+ aged general public,132 nations, 2007	Self-report on a single question: Do you feel your life has an important purpose or meaning?	Best-worst possible life	ZZ 2007	Oishi and Diener, 2014
18–88 aged, general public, Denmark, 1993	Single question: “Do you feel part of a larger whole?”	Happiness Life satisfaction Affect level	DK 1993	Ventegodt, 1995
50+ aged, general public, Europe, 2006,2007	How often do you feel that your life has meaning?	Life satisfaction	ZZ 2006	Becchetti et al., 2016
25+ aged, USA, 199?	3 questions on perceived meaning	Happiness Life satisfaction	US 1990	Ryff and Keyes, 1995
**Special publics**				
*Patients*				
Cancer out-patients, Warsaw, Poland, 2008	Purpose in life test	Best-worst possible life Life-satisfaction	PL 2008	Wnuk et al., 2012
18–42 aged mental patients, before and after psychotherapy, Netherlands, 199?	Life regard index framework	Happy person Life satisfaction	NL 1991	Debats, 1996
Remitted mental out-patients and controls, Italy, 199?	Purpose in life scale (Ryff)	Affect level	IT 1995	Rafanelli et al., 2000
Cancer patients, followed 6 months after onset, Trier, 198?	Self report on questions focusing on attempts to find meaning in the illness experience, especially with reference to religious issues	Affect level	XZ 1988	Filipp and Klauer, 1991
*Age groups*				
31–33 aged, Denmark 1993, born in University Hospital in Copenhagen	Do you think your work is meaningful?	Happiness Life satisfaction Affect level	DK 1993	Ventegodt, 1996
30–51 aged, urban areas in seven countries, 2006	questions about meaning associated with 10 domains of life	Life satisfaction	ZZ 2006	Delle-Fave et al., 2011
Elderly, British Columbia, Canada, 2005	How satisfied are you with your sense of meaning of life?	Life satisfaction Happiness	CA BC 2005	Michalos et al., 2007
50+ aged, general public, Europe, 2006, 2007	How often do you feel that your life has meaning?	Satisfaction with life	ZZ EU 2006	Becchetti et al., 2016
Students, Turkey, 2012	10 questions on perceived meaning	Satisfaction with life scale Affect balance	TR 2012	Dogan et al., 2012
147 college students, Philippines 201?	Five questions on perceived meaning and purpose	Affect balance	PH 2012	Navares, 2017
College students, USA, followed seven weeks 1991–1992	Three questions on perceived meaning and purpose	Average mood over 52 days	US 1991	Diener et al., 2012
18–46 aged undergraduate psychology students, Netherlands, 198?	Life regard index	Happy person Satisfaction with life	NL 1985	Debats, 1990
Aged 17–40, undergraduate university students, Canada, 2017	Self report on “functioning well” on 8 items	Affect balance	CA 2017	Passmore et al., 2018
University students, Great Britain, 1995	Purpose in Life test	Affect balance	GB1995/1	Lewis et al., 1997
*High school pupils, age 15*				
Albania, 2018	Three questions on perceived	Satisfaction with life	AE 2018	OECD, 2019
Argentina, 2018	meaning and purpose	Affect balance	AL 2018	
Austria, 2018			AR 2018	
Azerbaijan (Baku), 2018			AT 2018	
Belarus, 2018			BY 2018	
Bosnia Herzegovina, 2018			BA 2018	
Brazil, 2018			BR 2018	
Brunei Darussalam, 2018			BN 2018	
Bulgaria, 2018			BG 2018	
Chile, 2018			CL 2018	
Colombia, 2018			CO 2018	
Costa Rica, 2018			CR 2018	
Croatia, 2018			HR 2018	
Czech Republic, 2018			CZ 2018	
Denmark, 2018			DK 2018	
Dominican Republic, 2018			DO 2018	
Estonia, 2018			EE 2018	
Finland, 2018			FI 2018	
France, 2018			FR 2018	
Georgia, 2018			GE 2018	
Germany, 2018			DE 2018	
Greece, 2018			GR 2018	
Hungary, 2018			HU 2018	
Iceland, 2018			IS 2018	
Indonesia, 2018			ID 2018	
Ireland, 2018			IE 2018	
Italy, 2018			IT 2018	
Kazakhstan, 2018			KZ 2018	
Lebanon, 2018			LB 2018	
Lithuania, 2018			LT 2018	
Luxembourg, 2018			LU 2018	
Morocco, 2018			MA 2018/1	
Malaysia, 2018			MY 2018	
Mexico, 2018			MX 2018	
Moldova, 2018			MD 2018	
Montenegro, 2018			ME 2018	
Netherlands, 2018			NL 2018	
North Macedonia, 2018			MK 2018	
Panama, 2018			PA 2018	
Peru, 2018			PE 2018	
Portugal, 2018			PT 2018	
Qatar, 2018			QA 2018	
Russia (Tatarstan), 2018			RU 2018	
Switzerland, 2018			CH 2018	
United Arab Emirates, 2018			AE 2018	
United Kingdom, 2018			GB 2018	
*Users of positive psychological interventions (PPIs)*				
Users of a positive psychology website, United States, 2002-2005	I am looking for something that makes my life feel meaningful. I am always looking to find my life's purpose I am always searching for something that makes my life feel significant. I am seeking a purpose or mission for my life. I am searching for meaning in my life.	Affect balance	US 2002	Park et al., 2010
Participants in a meaning training, Australia and New Zealand, 202?	In the past several hours I have led a purposeful and meaningful life Treated also answered questions - What activity are you currently doing? - Is your current activity fulfilling?	Affect balance Momentary life satisfaction	AU 2016	Van Agteren et al., 2021
*Further populations*				
Working population, Spain, 2012	Purpose in life	On the average, what percentage of the time do you feel?	ES 2012	Merino and Privado, 2015
65+aged, ethnic Korean naturalized as Japanese and Japanese, Japan, 2005	One question having sense of purpose in life (vs not)	Write any number between 0 and 100 that describes your quality of life	JP 2004	Moon and Mikami, 2007

### Description of research findings

**Figure 2:**
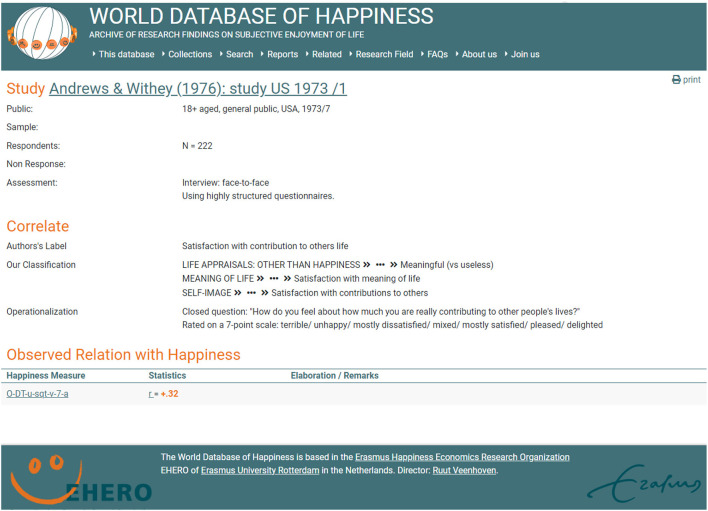
Example of a finding page. https://worlddatabaseofhappiness.eur.nl/studies/andrews-withey-1976-study-us-1973-1-228/. Source: World Database of Happiness. Reproduced with permission.

The findings obtained using a valid measure of happiness are described on electronic “finding pages”, using a standard format and terminology. Each page has a unique Internet address, to which we have linked in the text of this review. An example of such a finding page is presented in Figure 2.

### Format of this review

In this review, we started by summarizing the research findings in Table 2 in which the observed statistical relationships are presented in **+**, **–**, or 0 signs. These signs link to finding pages in the World Database of Happiness. If you click on a sign, one such finding page will open.

**Table 2:**
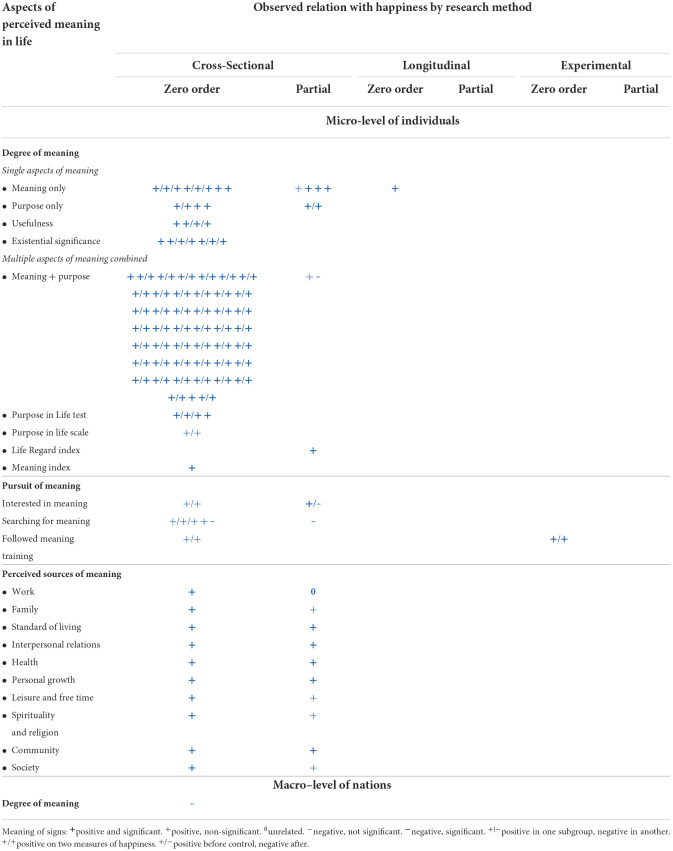
171 findings on the relation between perceived meaning of life and satisfaction with life.

Meaning of signs: ^**+**^positive and significant. ^+^positive, non-significant. ^0^unrelated. ^−^negative, not significant. ^**−**^negative, significant. ^+|−^positive in one subgroup, negative in another. ^+/+^positive on two measures of happiness. ^+/−^positive before control, negative after.

#### Organization of the findings

In Table 2 we first sorted the findings by the research method used and present these in three separate columns. We distinguished (1) cross-sectional studies, which asses same-time relationships between the perceived meaning of one's life and satisfaction with life, (2) longitudinal studies, which assess change in life satisfaction following change in meaning, and (3) experimental studies, which assess the effect on induced change in meaning on life satisfaction. In Table 2, we also distinguish between studies at the micro-level, which assess the relation between meaning and life satisfaction of individuals, and studies at the macro-level, which link average meaning in nations to average life satisfaction of citizens.

#### Presentation of the findings

The observed quantitative relationships between the perceived meaning of one's life and satisfaction with life are summarized in three possible signs: + for a positive relationship, – for a negative relationship, and 0 for a non-relationship. Statistical significance is indicated by printing the sign in **bold**. Each sign contains a link to a finding page in the World Database of Happiness on which the reader can find more detail.

Some of these finding pages appear in more than one cell of the tables. This is the case for pages on which both a “raw” (zero-order) correlation is reported and a “partial” correlation in which the effect of control variables is removed.

#### Advantages and disadvantages

The advantages of such representation are as follows: (1) an easy overview of the main trend in the findings, in this case, the many + signs, (2) access to full detail behind the links, (3) an easy overview of the white spots in the empty cells in the tables, and (4) easy updates, by entering new signs in the tables, possibly marked with a color.

The disadvantages are as follows: (a) that much detail is not directly visible in the + and – signs, (b) in particular not the effect size and control variables used, and (c) that the links work only in electronic texts.

This review technique has been applied in earlier syntheses of research on “Happiness and Private Wealth” (Jantsch and Veenhoven, 2019), “Happiness and Healthy Eating” (Veenhoven, 2021), and “Happiness and Consumption” (Veenhoven et al., 2021).

## Results

An overview of the 171 research findings is presented in Table 2. We can now inspect what these findings tell us about the correlation between the perceived meaning of life and satisfaction with life.

### Degree of perceived meaning in life and satisfaction with life

Most of the findings are on this topic. Correlations at the micro-level of individuals are presented in the upper part of Table 2 and one correlation at the macro-level of nations is at the bottom of this table.

#### Is there a correlation?

Yes, there is. Of the 171 observed relationships, only one found no correlation (0) and 15 studies observed a correlation that did not reach statistical significance. The other 155 findings denote a significant correlation.

#### Direction of the correlation

Plus signs (+) dominate in Table 2, denoting that the perceived meaning of life typically goes with greater satisfaction with life; however, there is a notable exception. Although correlations at the *micro-level of individuals* are positive, a study at the *macro-level of nations* found a negative correlation between the average degree of perceived meaning and average life satisfaction. We will discuss this phenomenon in section “Why a negative correlation at the macro level of nations between perceived meaning of life and average satisfaction with life?”

#### Strength of the correlations

A total of 136 findings are expressed in a comparable correlation coefficient. These, quite sizable, effect sizes are presented in Table 3. The average correlation between the perceived degree of meaning and life satisfaction at the micro-level is +0.36.

**Table 3:**
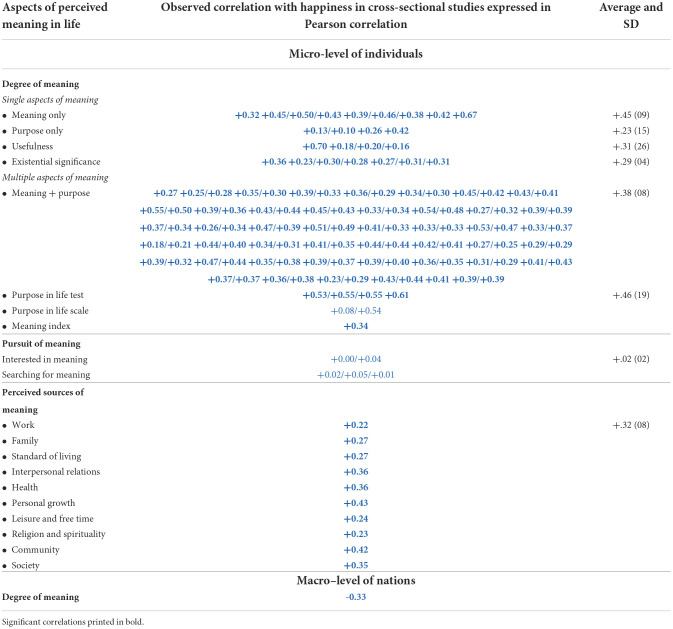
136 findings on strengths of correlation between perceived meaning of life and satisfaction with life.

Significant correlations printed in bold.

#### Variability across cultures

Since most correlations are positive, variability is in the size of the correlations. The OECD study among high school pupils covers 50 nations from different parts of the world. In Table 4, we marked the coefficients observed in different parts of the world using colors. No systematic difference appeared.

**Table 4:**
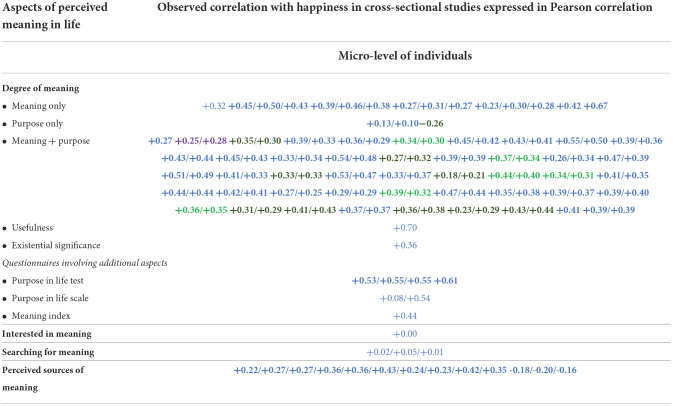
117 findings on strengths of correlation between perceived meaning of life and satisfaction with life in Africa, Asia, Latin America, Middle East, and Western nations (Australia, Europe, and North America).

#### Similarity across components of happiness

Likewise, we visualized a possible difference across components of happiness in Table 5, marking the correlations with overall life satisfaction as Blue, with affect level as Red, and with contentment as Purple. We also found no clear difference.

**Table 5:**
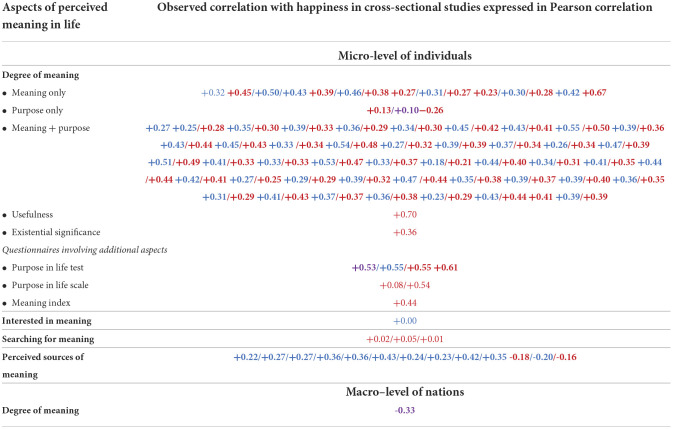
136 findings on strengths of correlation between perceived meaning of life and satisfaction with life distinguishing between overall life satisfaction, hedonic level of affect, and contentment.

#### Difference across questions on “meaning”, “purpose”, and “significance”

Most studies used questions on multiple aspects of the perceived meaning of life; only 25 of the 136 correlates in Table 3 are about the correlation between single aspects of perceived meaning and happiness. These correlates are presented in the top segment of Table 3. No great differences appeared, although the correlations with questions about “meaning” tended to be stronger than the correlations with questions about “purpose” and “existential significance”. We will come back to this difference in “How does satisfaction with life affect perceived meaning of life?”

### Pursuit of meaning and satisfaction with life

The findings on this matter are reported in the middle rows of Table 3. *Interest* in the meaning of one's life appears to be unrelated to life satisfaction. At first sight, the reported *pursuit* of meaning was found to be unrelated to life satisfaction. After controlling for the presence of meaning, the correlation became negative. A comparison across nations also showed a negative correlation in rich countries but a positive correlation in poor countries. We will come back to this latter phenomenon in section “Why a negative correlation at the macro level of nations between perceived meaning of life and average satisfaction with life?”

### Perceived sources of meaning and satisfaction with life

The findings on this subject are reported in the lower part of Table 3. All the correlations were positive, but there was a difference in strengths. Seeing meaning in social bonds was most strongly related to life satisfaction, as in the cases of “interpersonal relations”, “community”, and “society”. The low correlation with seeing meaning in “family” does not fit this pattern. Life satisfaction is related equally strongly to seeing meaning in life itself, such as in the cases of “health” and “personal development”. The weakest correlations were with seeing meaning in “spirituality and religion” and with “work”.

## Discussion

What do these correlational findings tell us about the causal interplay between the perceived meaning of one's life and satisfaction with life?

### Is there an innate need for meaning?

At first sight, the findings fit the theory that we have an innate need for meaning (cf. section “Questions on correlation”) and that therefore, the more meaning we see in our life, the more satisfied we are with it. Not only are the correlations positive and sizable as we saw in Table 3, but they also appear to be universal, given the little variation across countries seen in Table 4. The strong correlations with affect level, which we saw in Table 5, can also be interpreted as resulting from need gratification, especially in the context of Veenhoven's (2009) theory of happiness in which the affective component reflects the gratification of universal human needs.

However, we also met with a finding that contradicts this theory. A lot of people appear not to care about the meaning of their life and are still reasonably happy. This was observed in the study of DeHart et al. (2022) among the general public of the Netherlands, where 20% agreed with the statement “the meaning of life is a subject that does not interest me very much”. Although marginally less happy than their fellow citizens who disagreed with this statement, these people were still quite happy with an average of 7.47 on the 0–10 scale of life satisfaction. So, the quest for seeing meaning in one's life is not universal and not very pressing.

At a more theoretical level, one can also doubt that there is an innate need for seeing meaning in one's life. A “need” is not just a “want” or a “preference” but something that is required for survival and which has, for this reason, become an innate part of human nature. This is the case for our needs for food and social belongingness and can also be seen to apply to the need to use and develop our potentials. The survival value of the perceived meaning of one's life is less obvious, in particular when situated in the life situations of our early forefathers. Because of their survival value, needs were linked with strong affective signals. The affective signals that attend the perceived meaning of one's life are typically less strong than those of hunger and loneliness.

An alternative explanation for the universal quest for meaning is that it is a consequence of human cognition, self-awareness in particular. Because we know that we are, we tend to wonder why we are and whether our life serves any other good beyond our own life. Although these questions come to mind, we can live without convincing answers. Seen in this light, the quest for meaning can be better seen as an evolutionary unintended by-effect of the otherwise highly functional capacity of cognition. This interpretation fits the distinction Wentholt (1995) makes between innate “organic needs” which we share with most primates and “universal strivings” which come forth from human self-awareness.

### How does perceived meaning of life otherwise affect satisfaction with life?

If not automatically in response to the gratification of an innate need, how else can seeing the meaning of life contribute to satisfaction with life? One possibility is that a sense of meaning is pleasant, even if not required. In this respect, the perceived meaning of life is comparable to the enjoyment of arts, not a basic need either, but even so a source of satisfaction.

In this view, the perceived meaning of one's life is one of the appraisals of life aspects that contribute to one's satisfaction with life as a whole, and as such is comparable to the perceived “richness of life”, which also goes with greater satisfaction with life (Oishi and Westgate, 2021). In section “Questions on correlation”, we depicted this mechanism as a “bottom-up” effect.

A related effect seems to be that a sense of meaning can help us to cope with misery, a heuristic being “My life is full of suffering, but I live for a good cause”. In this way, a sense of meaning keeps us less unhappy than we would have been otherwise. This mental comfort can have a price when the quest for meaning leads to behaviors that undermine other sources of happiness, such as when one's health is sacrificed for a cause. In such cases, a sense of meaning can reduce happiness on the balance. This could be one of the reasons why the average sense of meaning tends to be higher in countries where average happiness is low, as we will discuss in more detail in section “Why a negative correlation at the macro level of nations between perceived meaning of life and average satisfaction with life?”

This brings us to the wider instrumental value of perceiving meaning in one's life, which positive psychologists typically see as a “strength”. In this view, a sense of meaning facilitates functioning by adding a moral premium to one's activities, which helps us to get involved and overcome dips. In that context, the main causal mechanism will be “activity”, also known as “fully functioning”. Activity appears to be the main determinant of life satisfaction, be it that more activity is not always better. We feel best at a personal optimum between boredom and anxiety (Csikszentmihalyi, 1995).

A sense of meaning can also affect life satisfaction more indirectly by fostering other mental strengths, such as your identity and self-esteem. It can also affect social conditions for happiness, such as your social prestige and in that way possibly marriage chances. Again, such effects will not always be positive.

### How does satisfaction with life affect perceived meaning of life?

The observed correlations between perceived meaning of life and satisfaction with life should not be interpreted too easily as a causal effect of the former on the latter, since reversed causality is likely to be involved in this case.

One causal mechanism is certainly that meaning of one's life is often seen in one's life as such. We saw in Table 3 that “health” and “personal development” are seen as sources of meaning. Even more, telling is the qualitative study done by Kok et al. (2015) among Malaysian youngsters, in which about half of the respondents appeared to see meaning in leading a happy life, thus implying a correlation with life satisfaction.

Another causal effect of life satisfaction on perceived meaning is found in Fredrickson's (2004) “Broaden and Build Theory” of positive affect, which draws on a large body of empirical research. When we feel good, our adaptational repertoire “broadens” in several ways: Good mood enhances activity and makes us more aware of what goes on in other people and will make us more creative in solving problems. This results in the long-term “building” of more resources, both career-wise and in personal relations. As such, life satisfaction adds to one's chance of doing meaningful things.

Apart from adding to the actual meaningfulness of one's life, life satisfaction will also affect your mere *perception* of how meaningful your life is. The meaningfulness of one's life is an intangible object, the perception of which is highly vulnerable for the observer's mental set. As such, it is likely that happy people tend to see more meaning in their lives than unhappy people, irrespective of the actual meaning of their lives. In this context, it is worth remembering Table 3, in which we see stronger correlations of life satisfaction with general statements of “meaning”, than for more specific aspects of meaning, such as “purpose” and “existential significance”.

A related effect seems to be that the issue of the meaning of one's life will present itself more urgently when one is unhappy and wonders “What is this suffering good for?” and “Why do I live?”. Since convincing answers to such questions are often not available, unhappy people tend to become more aware of a lack of meaning in their lives and some will attribute their unhappiness to this lack. This will also boost the correlation between the perceived meaning of life and satisfaction with life.

### The effect of perceived meaning on life satisfaction and of life satisfaction on perceived meaning of life

As yet, we lack data to provide an answer to this chicken and egg problem, all we can say is that the one experimental study, undertaken so-far, suggests that there is a causal effect of perceived meaning of life on satisfaction with life. This is the case of training in seeing meaning, where the experimental group gained more happiness right during the training than the control group. See column “experimental” in Table 2. Apart from the weaknesses of this experiment, this is not to say that there is no effect of life satisfaction on perceived meaning, nor that this effect is less strong.

This is worth further investigation, both for the sake of intellectual curiosity and for priority setting in therapy and education. If life satisfaction is the main determinant in this relationship, it is better to foster life satisfaction than to preach meaning. We expand further on this in section “Implications of the top-down effect”.

### Why a negative correlation at the macro-level of nations between perceived meaning of life and average satisfaction with life?

Although the perceived meaning of life has been found to relate positively to satisfaction with life at the micro-level of individuals, one study at the macro-level of nations found a negative relationship, with a correlation between average sense of meaning and average life satisfaction of −0.33.

Although counter-intuitive at first sight, this is not uncommon. A similar pattern is observed with religion, although religious people are typically happier than the non-religious, average happiness tends to be lower in the most religious countries of the present-day world (Berg and Veenhoven, 2009), even though in the unhappy-religious countries the most religious people are still the happiest.

An explanation of this phenomenon holds that the main function of religion is to cope with misery and that people, therefore, tend to be more religious in miserable nations, which are typically less developed nations. Note that the abovementioned study also found a negative correlation between the perceived meaning of life in nations and their economic development, in which the relationship was mediated by average religiousness (Oishi and Diener, 2014). Religion may reduce the pain of miserable conditions, but not enough to provide a satisfying life. This medicine may also be worse than the disease, such as when religion inhibits cultural modernization, which societal pattern appears to fit human nature better than the traditional orientations that were functional in the agrarian phase of the development of human societies (Veenhoven, 2010).

In this case of perceived meaning, a related explanation is that the question about the meaning of life presents itself more in miserable conditions, in which little meaning can be found in one's life, life being full of suffering. In such contexts, there is more demand for meaning beyond one's own life, such as “saving the country” or “spreading the gospel”. Cultures respond to this demand by providing ways to see meaning in misery and glorifying them. As in the case of religion, some ways to meaning can bring people “from the frying pan into the fire”, for example, in the case of drawing them into a holy war.

This explanation fits the micro-level finding that time spent thinking about the meaning of life relates positively to life satisfaction in poor countries but negatively in rich countries as can be seen here.

### Implications of the top-down effect

Satisfaction with life tends to foster a sense of meaning in one's life (cf. section “How does satisfaction with life affect perceived meaning of life?”), and this top-down effect should be acknowledged in programs that aim to promote meaningfulness, such as currently in moral education.

Over the ages, education has not only involved the passing of knowledge but also included “character building”, in the context of which much attention has been devoted to “moral education”. Parts of this, traditionally religious inspired, education lives on in present-day positive psychology, in positive education in particular, since there is a moral component in notions of “positive mental health”. In the contemporary post-modern climate, there is less emphasis on the passing along of particular norms and values but more attention on developing a personal moral orientation. A view on the meaning of one's life is part of such orientation, and consequently, training in seeing meaning has been developed, an example of which is found here.

While the emphasis in moral education is to provide examples of leading a just life, there is growing attention to the development of the strengths and skills needed to live a just life. This shift links up with the notion of “performance character” (Lickona and Davidson, 2005) and fits the focus on strengthening strengths in positive psychology. People perform typically better when feeling good and for that reason Lawton et al. (2021) plea for including student wellbeing as a goal in moral education.

Given the probable effects of life satisfaction on perceived meaning in life (cf. section “How does satisfaction with life affect perceived meaning of life?”), moral educators should consider fostering the life satisfaction of their students, including positive affect, which mental state is typically not cherished by moral educators. For this purpose, moral educators can draw on the rich research on happiness education from positive psychology (Bergsma et al., 2021).

## Conclusion

Seeing meaning in one's life tends to go together with greater satisfaction with that life, at least at the micro-level of the individual. At the macro-level of nations, it goes with lower average life satisfaction. The relation between the perceived meaning of life and satisfaction with life is bi-directional and involves several causal mechanisms. An innate need for meaning is unlikely to be involved.

## Data availability statement

The empirical findings presented in this study are taken from referred research reports and summarized on electronic finding pages in the World Database of Happiness. Finding pages have a unique internet address to which links are given in this text. This text provides also links to excerpts of the studies concerned. This new technique of an online ‘finding archive' is described in this text. Further inquiries can be directed to the corresponding author.

## Author contributions

YS, YE, and RV were involved in the gathering and description of the research findings. Most of the text was written by RV. All authors contributed to the article and approved the submitted version.

## Footnotes

^1^(+) signifies the positive emotions, and (–) signifies negative emotions.
